# Morphometrıc evaluatıon of patıents wıth mandıbular hypomobılıty due to coronoıd process hyperplasıa usıng cone beam computed tomography

**DOI:** 10.1007/s00276-026-03830-0

**Published:** 2026-02-11

**Authors:** Şeyda Kurt Akgül, Ömer Faruk Boylu

**Affiliations:** Department of Oral and Maxillofacial Surgery, Faculty of Dentistry, Bolu Abant İzzet Baysal University, Bolu, Turkey

**Keywords:** Mandible, Hypomobility, Coronoid process, Hyperplasia, Temporomandibular joint

## Abstract

**Purpose:**

Mandibular hypomobility is a condition in which the jaw joint is restricted due to various causes, including temporomandibular joint disorders (TMD), ankylosis, head and neck infections and tumors, coronoid processes hyperplasia (CPH), and trismus. The aim of this study is to evaluate the relationship between the coronoid processes (CP) of patients diagnosed with CPH and other anatomical structures and to investigate how this affects temporomandibular hypomobility.

**Methods:**

The study included cone beam computed tomography (CBCT) images of 10 patients diagnosed with mandibular hypomobility due to bilateral CPH and 30 healthy individuals who underwent CBCT for other reasons (implant placement, impacted teeth, etc.). CBCT sections were used to measure condyle length (H1), CP length (H2), CP length over the zygomatic arch (H3), ramus length (H4), horizontal distance between CP and zygomatic arch (D), and the angle between CP and the mandibular plane (MP).

**Results:**

A statistically significant difference was found between the mean Cr-MP angles on the right and left sides in the study group (*p* = 0.031). Significant differences were observed between the study and control groups in H2, H2/H1, H3, H3/H2, H2/H4, and D distance measurements (*p* < 0.001). Additionally, a significant difference in the CP-MP angle was found between the groups (*p* = 0.003).

**Conclusions:**

Our study demonstrates that, in addition to CP length, factors such as the H2/H1 ratio, CP-MP angle, D distance, and H3 also influence mouth opening restriction.

## Introductıon

There are multiple causes of mandibular hypomobility, including TMD, facial space infections, CPH, fibrosis following radiation therapy, and head and neck tumors [11]. CPH is a rare congenital or developmental condition characterized histologically by abnormal elongation of the bone [19, 21, 23]. It can be unilateral or bilateral, with bilateral cases being more common [4, 23]. The primary clinical finding is painless, mechanically restricted mouth opening. The restriction occurs due to the elongation of the CP impinging on the frontal or medial surface of the zygomatic bone [3, 16]. Clinical examination should raise suspicion of CPH in patients experiencing difficulty in mouth opening or complaints of crepitation and pain in the zygomatic region without temporomandibular joint (TMJ) or masticatory muscle disorder symptoms [13, 16]. Unilateral CPH may also present with facial asymmetry and deviation towards the affected side [23]. While the etiology of CPH remains unclear, trauma, increased activity of the temporalis muscle, hormonal/genetic factors, and TMD are among the suggested causes [13, 20, 24]. In addition to panoramic radiographs, CBCT provides detailed imaging for evaluating CPH [2]. In our study, morphometric analyses of the coronoid processes were performed in patients diagnosed with mandibular hypomobility due to CPH. Additionally, we aimed to compare the coronoid process anatomy of diagnosed patients with that of healthy individuals using CBCT and to investigate the anatomical differences contributing to mouth opening restriction.

## Materıals and methods

CBCT images of 10 patients diagnosed with temporomandibular hypomobility due to bilateral CPH and 30 healthy individuals who underwent CBCT for other reasons (e.g., implants, impacted teeth) between July 2022 and July 2023 at the Department of Oral and Maxillofacial Surgery, Faculty of Dentistry, Bolu Abant Izzet Baysal University, were analyzed. The CBCT sections were examined in axial, coronal, and sagittal planes. Measurements were taken bilaterally and recorded along with the patients' gender and age. The relationship between the CP and other anatomical structures in CPH patients was evaluated to determine the extent and mechanism of its impact on mandibular hypomobility. Morphometric differences in CP anatomy between CPH patients and healthy individuals were also assessed.

### Imaging and measurement procedures

CBCT images were retrospectively analyzed using i-CAT Vision (ver.1.9) software on a laptop (Lenovo Ideapad 330, 15.6-inch, 5th generation Intel® Core™ i5-8250U processor) under natural light. The images were examined in axial, coronal, and sagittal planes, and measurements were recorded in an Excel spreadsheet. In sagittal sections, the mandibular ramus was aligned parallel to the sagittal plane. In axial and coronal sections, the midline was standardized by aligning the anterior nasal spine with the posterior nasal spine. Since i-CAT Vision software does not allow angle measurements, Weasis DICOM Medical Viewer (ver 4.2.0) software was used to determine angular values in CBCT images.

### Measurements and reference points

**Fig. 1:**
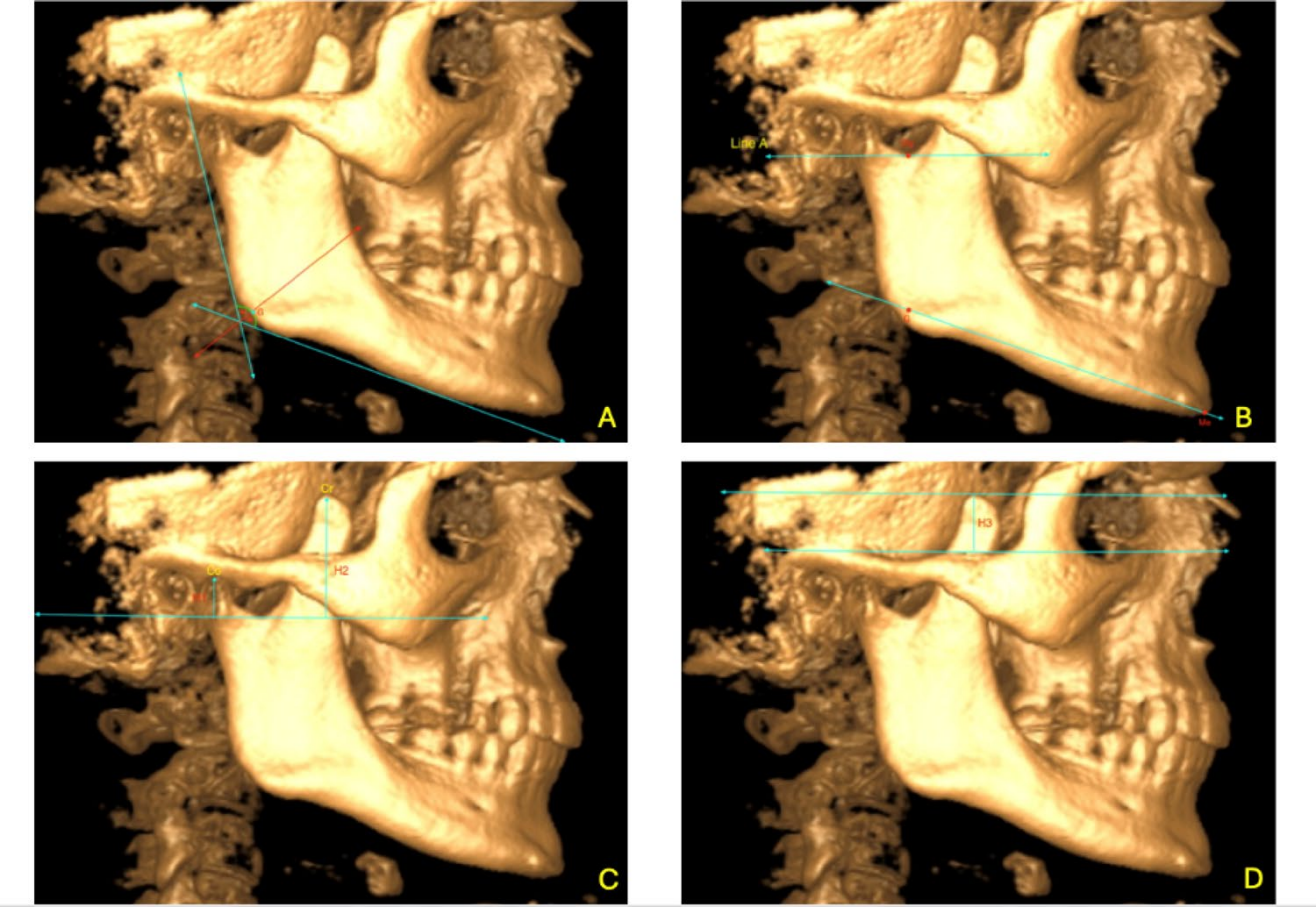
Sagittal and coronal views of the CBCT scans (**a** Gonion point, **b** Sig, Go, Me points and Line A, MP line, **c** Condylar length (H1), coronoid length (H2), point B-C, **d** Coronoid length (H3) remaining on the zygomatic arch point and Line A are shown)

**Fig. 2:**
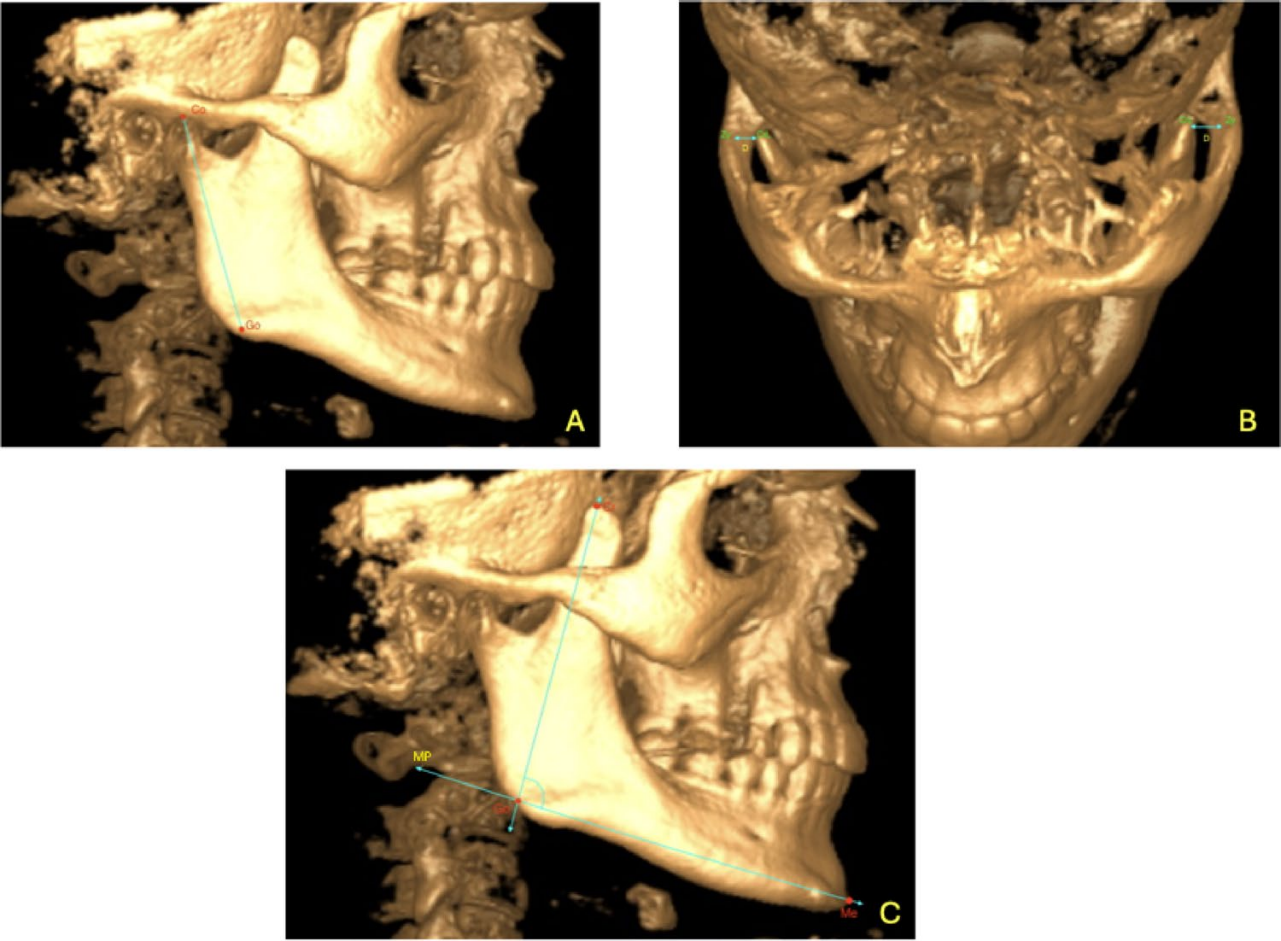
Sagittal and axial views of the CBCT scans (**a** Ramus length (H4), **b** Distance between the coronoid process, and the zygomatic arch (D), **c** Angle between the coronoid process and the mandibular plane (Cr-MP))

- **Co**: The highest point of the condyle
- **Cr**: The highest point of the coronoid process
- **CrL**: The most lateral point of the coronoid process
- **Zyg**: Zygomatic arch
- **T**: The peak point of the zygomatic arch
- **Sigmoid Notch (Sig)**: The concave region of the ramus between the condyle and coronoid process (Fig. 1b)
- **Gonion (Go)**: The point where the bisector of the angle formed by the tangent lines drawn to the posterior border of the mandibular ramus and the inferior border of the mandibular body intersects the mandibular outer contour (Fig. 1a and b)
- **Menton (Me)**: The lowest point of the mandibular symphysis contour (Fig. 1b)
- **Mandibular Plane (MP)**: The line passing through the Gonion and Menton points (Fig. 1b)
- **Line A**: A line parallel to the ground passing through the deepest point of the sigmoid notch in the sagittal section (Fig. 1b)
- **Condyle Length (H1)**: In the sagittal section, a perpendicular line is drawn from the highest point of the condyle (Co) to Line A (Point B). The distance between Co and Point B (mm) represents condyle length (Fig. 1c).
- **Coronoid Process Length (H2)**: In the sagittal section, a perpendicular line is drawn from the highest point of the coronoid process (Cr) to Line A (Point C). The distance between Cr and Point C (mm) represents coronoid length (Fig. 1c).
- **Coronoid Process/Condyle Ratio**: H2/H1
- **Coronoid Length Above the Zygomatic Arch (H3)**: In the coronal section, the distance (mm) between the peak of the zygomatic arch (T) and the highest point of the coronoid process (Cr) (Fig. 1d).
- **Coronoid Length Above the Zygomatic Arch/Coronoid Length Ratio**: H3/H2
- **Ramus Length (H4)**: In the sagittal section, the distance (mm) between the highest point of the condyle (Co) and the Gonion point, parallel to a tangent drawn along the posterior border of the mandibular ramus (Fig. 2a).
- **Coronoid Process Length to Ramus Length Ratio**: H2/H4
- **Distance Between the Coronoid Process and the Zygomatic Arch (D)**: In the axial section, the distance (mm) between the highest and most lateral point of the coronoid process (CrL) and the zygomatic arch (Zyg) (Fig. 2b).
- **Coronoid Process to Mandibular Plane Angle (Cr-MP)**: In the sagittal section, the angle between the line passing through the coronoid process (Cr) and the Gonion (Go) and the mandibular plane (MP) (Fig. 2c).

### Statistical analysis

The data were analyzed using IBM SPSS V23. The normality of the data distribution was assessed using the Shapiro-Wilk test. The paired samples *t*-test was used to compare normally distributed variables between the right and left sides, while the Wilcoxon test was applied for non-normally distributed variables. The Pearson correlation coefficient was used to analyze the relationships between normally distributed continuous parameters, whereas the Spearman’s rho correlation coefficient was used for non-normally distributed continuous parameters. For comparisons between two independent groups, the independent samples *t*-test was used for normally distributed variables, while the Mann-Whitney U test was used for non-normally distributed variables. The Fisher’s exact test was used to compare categorical variables between groups. Analysis results were presented as frequency (percentage) for categorical variables, and as mean ± standard deviation or median (minimum–maximum) for continuous variables. The significance level was set at *p* < 0.050.

## Results

The mean age of the study group was 35.1, while the mean age of the control group was 38.26. The gender distribution of the patients is shown in Table 1. In both the study and control groups, 90% of the participants were male, and 10% were female. Thus, there was no significant gender difference between the groups (*p* = 1.000) (Table 1).

**Table 1:** Comparison of gender by groups

	Group		
	Study group	Control group	p*
*Gender*			
Male	9 (90)	27 (90)	1.000
Female	1 (10)	3 (10)	

*Fisher's exact test

In Table 2, the parameters were compared by sides (right-left) in the study group. A statistically significant difference was found between the mean values of the coronoid process to mandibular plane (Cr-MP) angle (*p* = 0.031), while no significant differences were found in the other measurements. The mean value of the Cr-MP angle on the right side was 94.51, while on the left side, it was 91.68.

**Table 2:** Comparison of parameters by sides in the study group

	Side					
	Right		Left		Test statistic	*p*
	Mean ± standard deviation	Median (Min–Max)	Mean ± standard deviation	Median (Min–Max)		
H2	20.01 ± 4.91	18.75 (13.5–29.4)	21.6 ± 4.92	21.75 (15.9–29.4)	− 2.016	0.075*
H1	13.92 ± 2.43	14.1 (10.2–17.7)	15.18 ± 3.79	15 (9.9–20.4)	− 1.233	0.249*
H2/H1	1.52 ± 0.62	1.24 (0.97–2.61)	1.53 ± 0.66	1.27 (0.85–2.69)	− 0.459	0.646**
H3	2.5 ± 7.83	0.5 (− 6 to 16.2)	4.43 ± 8.49	2.79 (− 6 to 18.9)	− 1.785	0.108*
H3/H2	0.07 ± 0.33	0.04 (− 0.33 to 055)	0.16 ± 0.34	0.11 (− 0.3 to 0.64)	− 1.52	0.163*
H4	60.67 ± 5.66	61.55 (51.72–71.38)	61.67 ± 5.3	61,06 (51.47–70.65)	− 1.194	0.263*
H2/H4	0.33 ± 0.11	0.3 (0.21–0.56)	0.35 ± 0.1	0.34 (0.25–0.57)	− 1.23	0.219**
D	6.21 ± 1.91	6.51 (2.18–8.58)	6.78 ± 2.53	7.16 (3.08–10.6)	− 0.707	0.498*
Cr−MP angle	0.51 ± 7.91	93.9 (79.1–104.1)	91.68 ± 7.62	92.25 (75.6–103)	2.553	0.031*

*Paired *t*-test; **Wilcoxon test

Correlation analyses between coronoid process length and the D distance in the study group are presented in Table 3. A significant negative correlation was observed on the left side, whereas no significant correlation was detected on the right side.

**Table 3:** Relationship between coronoid process length and D distance in the study group

Side		D Distance	
		R	*p*
Right	Coronoid process length, H2	− 0.119	0.743
Left	Coronoid process length, H2	− 0.871	0.001

r: Pearson correlation coefficient; The relationship within each side has been examined

Correlation analyses in the study group are presented in Table 4. Significant correlations were observed only on the left side, with a positive association between coronoid process length and the coronoid process–mandibular plane angle and a negative association between the Cr–Zyg distance and the coronoid process–mandibular plane angle.

**Table 4:** Relationship between coronoid process length and coronoid process-MP angle in the study group for right and left side

Side	Variables		r	*P*
Right	Coronoid process length	H2	0.381	0.278
	Cr-Zyg distance	D	− 0.163	0.654
Left	Coronoid process length	H2	0.659	0.038
	Cr-Zyg distance	D	− 0.647	0.043

r: Pearson correlation coefficient; The relationship within each side has been examined

Inter-group comparisons between the study and control groups are summarized in Table 5. Coronoid process length (H2) and related proportional measurements, including the H2/H1 and H2/H4 ratios, were significantly higher in the study group than in the control group (*p* <0.001). Parameters describing the spatial relationship between the coronoid process and the zygomatic arch (H3 and H3/H2) also differed significantly between the groups (*p* <0.001).

**Table 5:** Comparison of parameters by groups

	Group				Test statistic	*p*
	Study group		Control group			
	Mean ± standard deviation	Median (Min–Max)	Mean ± standard deviation	Median (Min–Max)		
H2	20.81 ± 4.85	19.5 (13.5–29.4)	13.54 ± 3.77	12.15 (7.2–21.3)	153	< 0.001**
H1	14.55 ± 3.16	14.55 (9.9–20.4)	15.42 ± 2.94	15.9 (9–23.1)	− 1.123	0.265*
H2/H1	1.52 ± 0.62	1.27 (0.85–2.69)	0.9 ± 0.27	0.92 (0.33–1.45)	190	< 0.001**
H3	3.46 ± 8.01	0.5 (− 6–18.9)	− 8.5 ± 5.48	− 9.01 (− 18.9 to 8.4)	113.5	< 0.001**
H3/H2	0.11 ± 0.33	0.04 (− 0.33 to 0.64)	− 0.69 ± 0.5	− 0.6 (− 1.76 to 0.45)	6.624	< 0.001*
H4	61.17 ± 5.36	61.2 (51.47–71.38)	58.54 ± 5.21	59.47 (44.9–65.66)	461	0.122**
H2/H4	0.34 ± 0.1	0.31 (0.21–0.57)	0.23 ± 0.06	0.23 (0.11–0.35)	191.5	< 0.001**
D	6.49 ± 2.2	6.77 (2.18–10.6)	8.41 ± 1.82	8.66 (4.17–12.4)	− 3.865	< 0.001*
Cr-MP angle	93.1 ± 7.7	92.85 (75.6–104.1)	87.22 ± 7.38	86.65 (71.4–104.7)	3.051	0.003*

*Independent Two-Sample *t*-test; **Mann–Whitney U test

The coronoid process–mandibular plane (Cr–MP) angle was significantly higher in the study group compared with the control group (*p* = 0.003), whereas the horizontal distance between the coronoid process and the zygomatic arch (D) was significantly shorter in the study group (*p* <0.001).

In contrast, no statistically significant differences were observed between the groups with respect to condylar length (H1) or ramus length (H4).

## Dıscussıon

Mandibular hypomobility; autoimmune diseases such as scleroderma, syndromes like Plummer-Vinson and Treacher Collins, collagen disorders like oral submucous fibrosis, radiation therapy to the head and neck area, dental infections like pericoronitis, or infections of the salivary glands such as mumps, infections that affect the masticatory muscles and cause trismus, disc displacements, ankylosis, osteoarthritis, and TMD like CPH can lead to mandibular hypomobility [8, 11]. Therefore, it can lead to health problems such as chewing disorders, speech difficulties, poor oral hygiene, and malnutrition [8]. One of the causes of mandibular hypomobility, CPH is histologically defined as abnormal growth of the healthy CP [4]. The prevalence of the rare condition CPH is still debated. Annika Isberg examined 163 patients with restricted mouth opening and reported that in 8 of them (a 5% prevalence rate), the cause of the restricted mouth opening was CPH [10]. In a review involving 82 articles covering 115 cases, the average age of patients with CPH was reported to be 22.64 [7]. In another review analyzing 61 cases, the average age of patients with a diagnosis of CPH was stated to be 23 [23]. In this study, the average age of the 10 patients with mandibular hypomobility due to CPH was 35.1 (Table 1). We believe that the higher average age in our study for patients diagnosed with CPH is due to the failure to provide a timely and accurate diagnosis to the patients.

Annika Isberg examined 163 patients with restricted mouth opening and reported that the male-to-female ratio was 5:1 [10]. In a systematic review that included a total of 82 articles covering 115 cases, it was concluded that the male-to-female ratio in patients with CPH was 5:1 [7]. In another review analyzing 61 cases, the male-to-female ratio in patients with a diagnosis of CPH was reported to be 3.3:1 [20]. In this study, which included 10 patients with mandibular hypomobility due to CPH, the male-to-female ratio was found to be 9:1, and similar results were obtained with the literature, showing that CPH is more common in males.

In a study by Nayak et al. [18] on 160 dry human mandibles, it was reported that the coronoid process length ranged from 13.9 to 15.3 mm, with the right side being longer. Chauhan et al. [6] reported the coronoid process length in dry mandibles with a diagnosis of CPH as 24 mm on the right side and 26 mm on the left side. In the H2 measurements performed in our study group, the average on the right side was found to be 20.01 mm, and the average on the left side was 21.60 mm (Table 2). The anterior part of the temporal muscle attaches to the coronoid process and is active during maximal teeth clenching. Bone formation in the coronoid process may increase depending on the activity of the temporal muscle, which may lead to an increase in the process length [22]. We believe that the longer coronoid process on the left side may be due to the patients' muscle activity or left-sided chewing habits.

Tavassol et al. [20] observed a significant difference when comparing the coronoid process length of 40 control patients (20 adults, 20 children) with that of a 13-year-old patient diagnosed with CPH. The average coronoid process length in the control patients was 13.02 mm in adults and 12.43 mm in children, whereas the coronoid process length of the patient with CPH was recorded as approximately 20.0 mm. In a retrospective study where the coronoid process height of four male patients diagnosed with CPH was evaluated using 3D CT and compared with 10 male control patients, the average coronoid process height in the control patients was found to be 19.9 mm, while in the CPH patients, it was 24.75 mm [15]. In our study, the average H2 value was measured as 20.81 mm in the study group and 13.54 mm in the control group, showing a statistically significant difference between the groups (*p*<0.001) (Table 5). Bilgili et al. [5] in a study where 32 unilateral CPH cases were examined using CBCT, retrospectively evaluated the vertical lengths of the mandibular condyles on both the affected and unaffected sides. On the side with unilateral CPH, the average condylar height was reported as 13.23 mm, while on the unaffected side, it was 15.32 mm. In our study group, the average right condylar height was 13.92 mm, and the average left condylar height was 15.18 mm, with no significant difference observed between the right and left sides. The average condylar length in patients diagnosed with CPH in our study was 14.55 mm, while the average condylar length in the control group was 15.42 mm, but no statistically significant difference was found (Table 4).

Kubota et al. [14] reported that if a patient complains of limited mouth opening and the coronoid-gonion to condyle-gonion ratio is greater than 1.1, further investigations are required for a definitive diagnosis of CPH. In our study group, the H2/H1 ratio was found to be 1.52 on the right side and 1.53 on the left side. Our patients were diagnosed with bilateral CPH, and the ratios we found are consistent with the literature. When comparing the groups in our study, the H2/H1 ratio was 1.52 in the study group and 0.9 in the control group, showing a statistically significant difference (*p*<0.001) (Table 5). Our results are in agreement with the literature.

Munk et al. [17] when evaluating the coronoid process lengths of three patients diagnosed with CPH using CT, found that in all three cases, the coronoid process exceeded the inferior edge of the zygomatic arch. In one case report, a coronoid process was found to be at least 1 cm above the inferior edge of the zygomatic arch [12] In another study, the coronoid process length over the zygomatic arch of a male patient with Jacob's disease was measured using 3D CT, and the value was reported to be 1.3 cm [1]. Studies have reported that the coronoid process apex being at the level of the zygomatic arch is normal. However, when the apex of the coronoid process is located at least 1 cm above the inferior edge of the zygomatic arch, CPH is mentioned [5]. In our study, the coronoid process length over the zygomatic arch was measured as 2.5 mm on the right side and 4.43 mm on the left side (Table 2). The presence of bilateral CPH in our patients is supported by the literature. In our study, this value was 3.46 mm in the study group and − 8.5 mm in the control group, showing a statistically significant difference between the groups (*p*<0.001). Our study supports the literature.

In our study, when evaluating the H3 to H2 ratio, we found that in the study group, it was 0.11 mm, and in the control group, it was − 0.69 mm, with a statistically significant difference between the groups (*p*<0.001). The values for H3 and H2 related to CPH in the study group were higher compared to the control group. Therefore, we believe that the high H3/H2 ratio observed in our study is significant (Table 5).

In this study, when evaluating the ratio of H2 to H4, no significant difference was found between the right and left sides in the study group. However, the H2/H4 ratio was found to be 0.34 in the study group and 0.23 in the control group, with a statistically significant difference between the groups (*p*<0.001). We believe that the higher H2 height in the study group contributed to the higher H2/H4 ratio compared to the control group (Table 5).

In a case series of patients diagnosed with CPH, the distance between the coronoid process and the inner surface of the frontal part of the zygomatic bone was measured in axial CT scans. It was reported that a shorter distance could contribute to restricted mouth opening [9]. In our study, based on the established references, the D distance in the study group was found to be 6.21 mm on the right side and 6.78 mm on the left side. The average D distance in the study group was 6.49 mm, while in the control group it was 8.41 mm, with a statistically significant difference between the groups (*p* < 0.001) (Table 5). The shorter D distance in the study group compared to the control group supports the findings of Ilguy et al. [9] suggesting that this could contribute to mandibular hypomobility. However, the literature does not provide a clear value for the distance between the coronoid process and the inner surface of the frontal zygomatic bone that would definitively cause mandibular hypomobility. Therefore, further research in this area is needed.

In our study, we investigated the angle between the bilateral coronoid processes and the mandibular plane (Cr-MP). The aim of evaluating the Cr-MP angle was to determine whether smaller angles indicated the presence of more anteriorly positioned, tilted coronoid processes in relation to the mandibular plane. We hypothesize that an anteriorly positioned, tilted coronoid process is more likely to collide with the inner surface of the frontal part of the zygomatic bone, thereby contributing to restricted mouth opening. In a study, the angle between the mandibular plane and the coronoid process plane was compared in a male patient with Jacob’s disease with 30 patients who did not have TMJ complaints. No significant difference was observed between the two groups [1]. In contrast, in our study, the Cr-MP angle was found to be 94.51 degrees on the right side and 91.68 degrees on the left side, showing a statistically significant difference between the sides (*p *= 0.031). The Cr-MP angle in the study group was 93.1 degrees, while in the control group it was 87.22 degrees, with a statistically significant difference between the groups (*p *= 0.003) (Table 5). Despite the narrower angle in the control group, the coronoid process length and other measurements were found to be smaller compared to the study group. Therefore, we believe that there was no restriction in mouth opening in the control group. We suggest that further studies with larger sample sizes are needed.

In our study, we investigated the relationship between H2 and H1 in the study group. A statistically significant, high negative correlation was found between the right coronoid process length and the right condylar length (*p *= 0.031) (Table 3). The right coronoid process was measured to be shorter than the left coronoid process. However, the right condyle length was found to be shorter than the left condyle length, which is why we suggest a significant high negative correlation on the right side.

Additionally, a statistically significant negative relationship was observed between the coronoid process length and the coronoid process-zygomatic arch distance (CrL-Zyg, D) in the study group, as well as between the left coronoid process length and the left D distance (*p *= 0.001) (Table 3). As the left coronoid process length increased, the left D distance decreased. Ilguy et al. [9] mentioned that a shorter coronoid process-zygomatic arch distance could lead to restricted mouth opening. In our study, in patients with CPH-related mandibular hypomobility, an increase in coronoid process length was associated with a decrease in the D distance. More studies are needed to determine whether the coronoid process-zygomatic arch distance alone causes restricted mouth opening.

The relationship between coronoid process length and the Cr-MP angle was also evaluated in the study group. A statistically significant positive high correlation was observed between the left coronoid process length and the left Cr-MP angle (*p *= 0.038) (Table 4). As the left coronoid process length increased, the left Cr-MP angle also increased. We hypothesize that an increase in the Cr-MP angle, along with the coronoid process length, could contribute to mandibular hypomobility. However, studies with larger sample sizes are needed to confirm this.

Finally, we examined the correlation between the D distance and the Cr-MP angle in the study group. A statistically significant negative high correlation was found between the left D distance and the left coronoid process-MP angle (*p *= 0.043) (Table 4). As the left D distance increased, the left angle value decreased.

These findings suggest that changes in the coronoid process and distances related to the zygomatic arch and mandibular plane could be contributing factors in mandibular mobility. However, further research with larger sample sizes is necessary to draw more definitive conclusions.

The diagnosis of CPH is often challenging, as it may be overlooked in patients presenting with limited mouth opening and no clear TMJ pathology. The morphometric parameters evaluated in this study may assist clinicians in considering CPH in the differential diagnosis of mandibular hypomobility. In particular, assessment of the coronoid/condyle ratio and the spatial relationship between the coronoid process and the zygomatic arch on routine radiographic examinations may support earlier suspicion and referral for CBCT. These findings may contribute to improved diagnostic accuracy and support imaging-based clinical decision-making.

### Limitations

This study has several limitations. The retrospective design and limited sample size may restrict the generalizability of the findings. The use of a single-center study population may further limit the applicability of the results to other populations. Although CBCT provides reliable three-dimensional morphometric data, functional parameters were not directly correlated with the imaging findings. In addition, the cross-sectional nature of the study precludes causal inference regarding the relationship between coronoid process morphology and mandibular hypomobility.

## Conclusion

This study provides a descriptive morphometric evaluation of CPH using CBCT. The findings suggest that, in addition to CP length, proportional, angular, and spatial parameters of the CP differ between patients with mandibular hypomobility and healthy individuals. These observations indicate that CBCT may be a useful imaging modality for detailed anatomical assessment in patients presenting with limited mouth opening.

## Data Availability

The datasets generated and/or analyzed during the current study are available from the corresponding author on reasonable request.
